# Stress-Induced C/EBP Homology Protein (CHOP) Represses MyoD Transcription to Delay Myoblast Differentiation

**DOI:** 10.1371/journal.pone.0029498

**Published:** 2011-12-29

**Authors:** Joel Alter, Eyal Bengal

**Affiliations:** Department of Biochemistry, Rappaport Institute for Research in the Medical Sciences, Faculty of Medicine, Technion-Israel Institute of Technology, Haifa, Israel; North Carolina State University, United States of America

## Abstract

When mouse myoblasts or satellite cells differentiate in culture, the expression of myogenic regulatory factor, MyoD, is downregulated in a subset of cells that do not differentiate. The mechanism involved in the repression of MyoD expression remains largely unknown. Here we report that a stress-response pathway repressing MyoD transcription is transiently activated in mouse-derived C2C12 myoblasts growing under differentiation-promoting conditions. We show that phosphorylation of the α subunit of the translation initiation factor 2 (eIF2α) is followed by expression of C/EBP homology protein (CHOP) in some myoblasts. ShRNA-driven knockdown of CHOP expression caused earlier and more robust differentiation, whereas its constitutive expression delayed differentiation relative to wild type myoblasts. Cells expressing CHOP did not express the myogenic regulatory factors MyoD and myogenin. These results indicated that CHOP directly repressed the transcription of the MyoD gene. In support of this view, CHOP associated with upstream regulatory region of the MyoD gene and its activity reduced histone acetylation at the enhancer region of MyoD. CHOP interacted with histone deacetylase 1 (HDAC1) in cells. This protein complex may reduce histone acetylation when bound to MyoD regulatory regions. Overall, our results suggest that the activation of a stress pathway in myoblasts transiently downregulate the myogenic program.

## Introduction

Satellite cells are the progenitors of adult skeletal muscle. These cells quiescently reside in a niche between the muscle fiber and the basal lamina. They become activated in response to damage or injury; some differentiate and fuse with existing myofibers, while others sustain satellite cells properties [1]. The myogenic regulatory factors (MRFs) function in the different stages of the “life cycle” of satellite cells. Myf5 is expressed in quiescent satellite cells and MyoD is the earliest to be induced when these cells are activated and enter the cell cycle [2], [3], [4]. Muscles from MyoD knockout mice are severely deficient of regenerative capacity after injury [5]. The absence of MyoD postpones the transition of satellite cells-derived myoblasts from proliferation to differentiation [6], [7], [8]. It is believed that a subset of activated satellite cells lose their capacity to express MyoD in order to preserve a pool of stem cells for subsequent muscle regeneration. It is not known which process triggers the loss of MyoD expression and the mechanism(s) involved are still obscure.

Recent studies which combined genome-wide chromatin binding and expression profiling of MRFs have identified many unexpected targets for these factors [9], [10]. Among these targets there were several transcriptional regulators that function in response to different types of stress. At least two transcription factors, ATF4 and XBP1 that control certain aspects of the unfolded protein response (UPR) were found to be induced by MRFs as part of the myogenic program [10], [11]. These results suggested that myoblasts may be subjected to and are likely to respond to endoplasmic reticulum (ER) stress during the differentiation process.

The unfolded protein response (UPR) is an evolutionarily conserved signaling pathway that is activated by perturbations in ER homeostasis [12]. In response to the accumulation of unfolded proteins in the ER, the rate of general translation is attenuated, the expression of ER resident protein chaperones is induced, the ER compartment proliferates and ER associated degradation (ERAD) is activated to eliminate the misfolded proteins. If the prosurvival efforts are exhausted, ER stress-related apoptosis commences. Three different transducers mediate UPR; Inositol-requiring enzyme 1 (IRE1), activating transcription factor-6 (ATF6) and protein kinase RNA (PKR)-like ER kinase (PERK).

ER stress and UPR were demonstrated to participate in physiological processes like cell differentiation and maintenance of cells whose functions include the production and secretion of proteins, such as immune cells, endocrine and paracrine cells, hepatocytes, chondrocytes and osteoclasts [13]. ER stress occurs in skeletal muscle under pathological conditions such as myotonic dystrophy and chronic muscle atrophy [14], [15]. Less is known of the roles of UPR in normal muscle development and muscle regeneration. Recent studies by Morishima and colleagues [16], [17] indicated that ATF6, and CHOP were induced during myoblast differentiation *in vitro.* They suggested that ER stress occurring during differentiation induced ATF6-mediated apoptosis of myoblasts [17]. Exposure of myoblast cells to artificial tunicamycin-induced ER stress entailed massive apoptosis of cells, but also significantly elevated the efficiency of differentiation of the surviving cells [16].

In the present study we investigated the involvement of CHOP in the process of myoblast differentiation. We report that transient activation of stress-response proteins is intrinsic to myoblast differentiation program. In investigating the role of CHOP, we unexpectedly found that its transient expression in a subset of cells prevented their differentiation by repressing the transcription of *myod*. Our results indicate that CHOP binds to upstream transcription regulatory regions of *myod* thereby repressing its transcription. Taken in sum, these findings indicate that CHOP expression is induced in myoblasts to prevent their premature differentiation.

## Results

### Stress markers are transiently induced during myoblast differentiation

**Figure 1:**
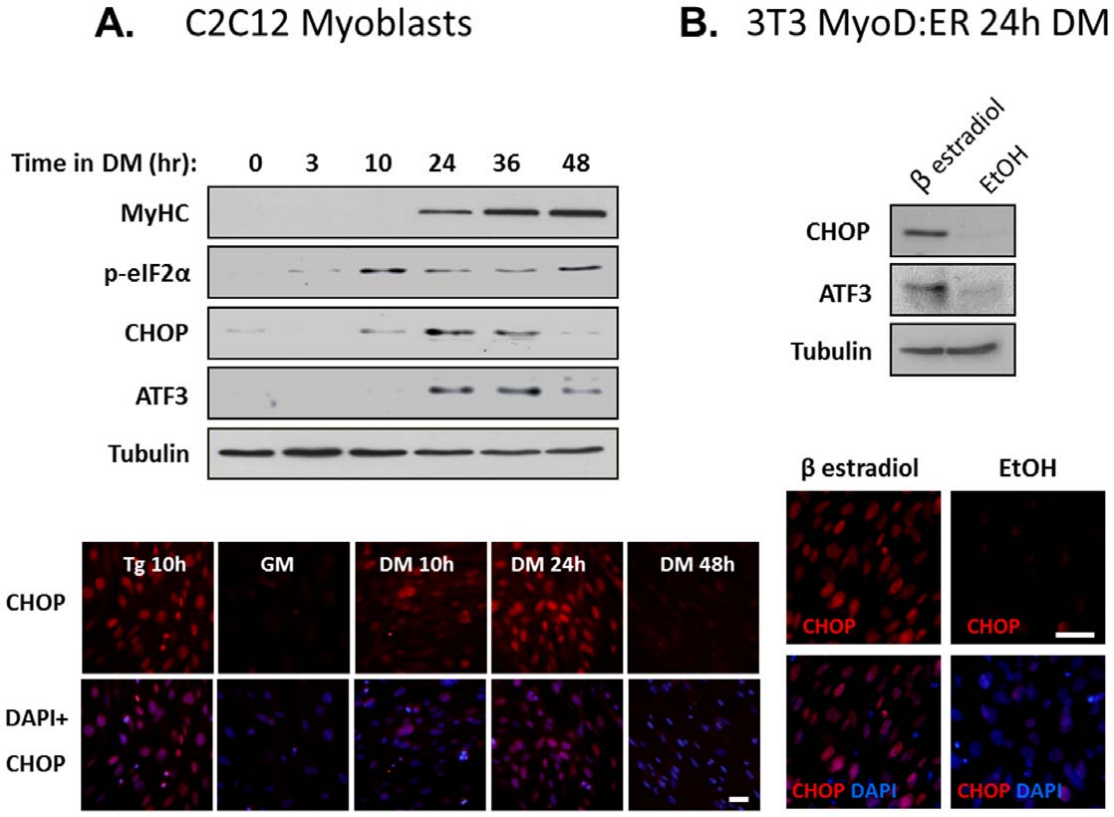
Transient expression of stress response proteins during myoblast differentiation. (A) C2C12 myoblasts were differentiated in DM and proteins were extracted at different time points as indicated. Protein were separated and analyzed by Western blotting (Upper panel). In another experiment C2C12 myoblasts were differentiated as is described. Cells were fixed and analyzed by immunostaining with an antibody to CHOP. CHOP in red, DAPI in blue (Lower panel). Bar, 50 µm. (B) 3T3 MyoD:ER cells were differentiated in DM containing ethanol or β estradiol (0.1 µM) for 24 hours. Proteins were extracted and were analyzed by Western blot with the indicated antibodies (Upper panel). Cells were fixed and were analyzed by immunostaining with an antibody to CHOP. CHOP is red, DAPI in blue (Lower panel). Bar, 50 µm.

Morishima and colleagues [17] reported that the ER stress sensor ATF6 was specifically activated in myoblasts undergoing apoptosis. Interestingly, CHOP, another downstream UPR effector was also activated, and was expressed in surviving myoblasts. To investigate a possible role of CHOP during muscle differentiation, we monitored the expression of several stress markers at several time points after inducing the differentiation of C2C12 myoblasts. Our results show that phosphorylation of eIF2α was initiated after 3 hours of myoblast growth in differentiation medium (DM) and the expression of CHOP and ATF3 transcription factors was induced after 10 and 24 hours, respectively (Figure 1A). Expression of the two transcription factors was transient and it diminished at 48 hours. Overall, the expression of stress markers preceded terminal differentiation (data not shown). Detection of CHOP by immunostaining indicated that it was localized in the nuclei of cells growing in DM (Figure 1A). However, it was expressed in many but not in all cells. The extent of CHOP expression in differentiating myoblasts was comparable to its expression following treatment of myoblasts with tapsigargin, an ER stress inducer. Next, we asked whether the induced expression of stress proteins was a general response of cells to the serum starvation conditions that were required the initiation of the differentiation process. For this purpose, we took advantage of a fibroblast cell line expressing a MyoD-estrogen receptor fusion protein (3T3 MyoD:ER) [18]. These cells grow as fibroblasts with MyoD:ER residing in the cytoplasm. When β estradiol is added to the medium, it induces nuclear translocation of the MyoD:ER chimera thus turning cells into myoblasts. We observed that serum starvation (DM) induced CHOP and ATF3 expression only in those cells treated β estradiol but not in cells treated with its solvent ethanol (Figure 1B). We conclude, therefore, that serum starvation induces CHOP and ATF3 expression in myoblasts but not in fibroblasts.

### Phosphorylation of eIF2α is necessary for the expression of CHOP and ATF3

**Figure 2:**
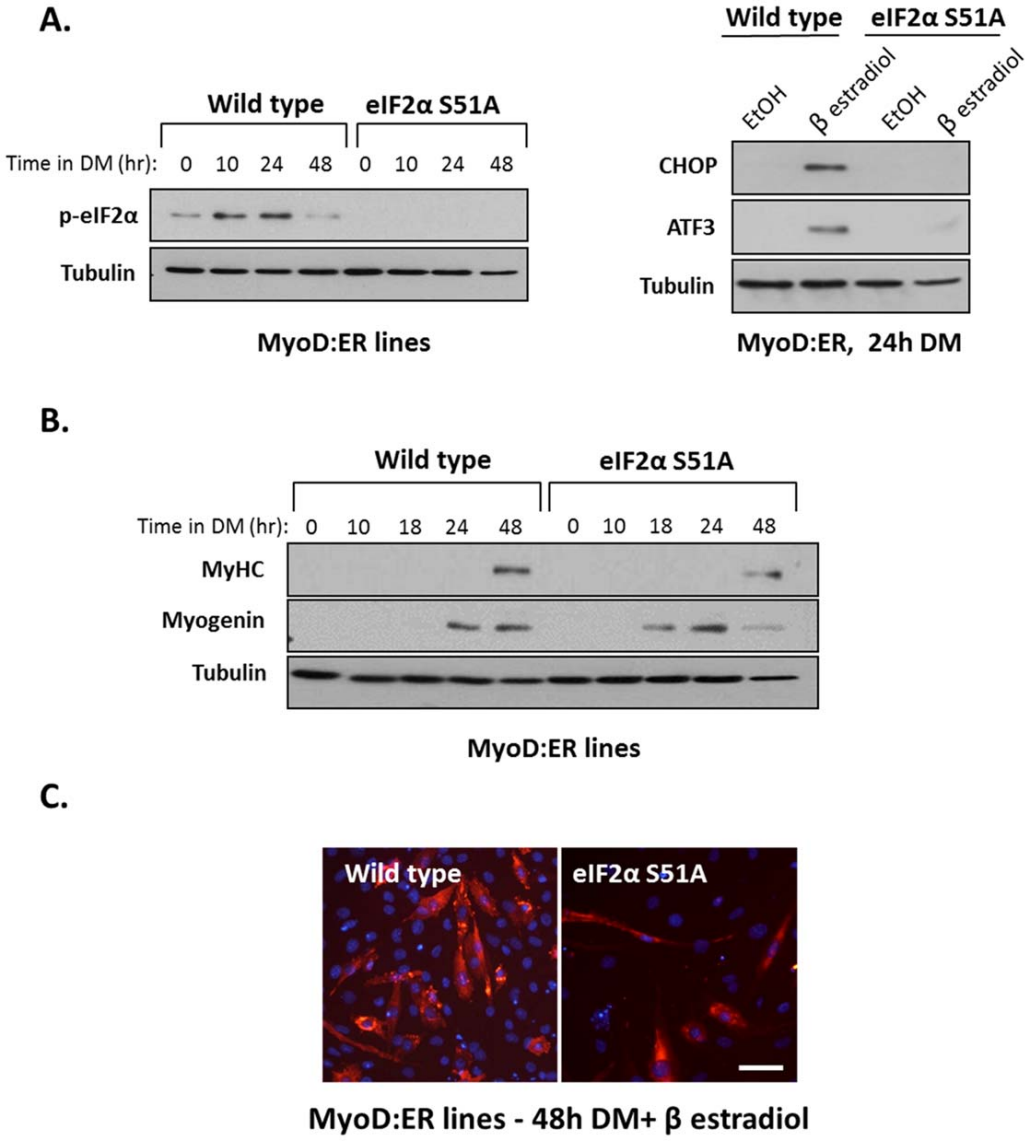
Muscle differentiation of eIF2αS51A knockin cells. Wild type eIF2α and mutated eIF2αS51A fibroblasts were infected with viruses encoding MyoD:ER protein. (A) Cells were allowed to differentiate in DM and β estradiol (0.1 µM) for the indicated time periods and proteins were analyzed by Western blot (left panel). Cells were grown in DM and ethanol or β estradiol (0.1 µM) for 24 hours and CHOP and ATF3 proteins were analyzed by Western blot (right panel). (B) Cell lines were grown as is described in A, and were analyzed by Western blot. (C) Cell lines were grown in DM for 48 hours. Cells were immunostained with an anti MyHC antibody (MF20); MyHC in red, DAPI in blue. Bar, 50 µm.

Transient phosphorylation of eIF2α at serine 51 indicates that protein synthesis may be attenuated in cells expressing phosphorylated eIF2α. Indeed, pulse-labeling of proteins in myoblasts at different growth periods in DM indicated that protein synthesis was transiently reduced during differentiation (Figure S1). We asked whether the expression of CHOP and ATF3 was induced subsequent to the phospohrylation of eIF2α. Embryonic fibroblasts from the eIF2αS51A knockin mouse (S51A, unphosphorylatable mutant of eIF2α) and from wild type mouse were infected with a MyoD:ER-encoding virus [19]. As described above, these cells become myoblasts following treatment with β estradiol. First, we confirmed that the mutated eIF2α (S51A) was not phosphorylated under conditions that induced the phosphorylation of wild type eIF2α (Figure 2A, upper panel). Next, expression of CHOP and ATF3 was examined in cells that had either wild type or mutant eIF2α. We find that CHOP and ATF3 proteins were expressed in wild type cells, but not in mutated eIF2αS51A cells (Figure 2A, lower panel). This result indicated that the expression of the two stress proteins was dependent on the phosphorylation of eIF2α. The expression of the myogenic markers myogenin and myosin heavy chain (MyHC), indicated that both cell lines underwent differentiation in DM (Figure 2B). However, the expression of myogenin occurred earlier in eIF2αS51A cells than in wild type cells (Figure 2B). Immunostaining of MyHC confirmed that both cell lines formed myotubes, though the number of nuclei was significantly reduced in the mutant cells relative to wild type cells (Figure 2C). The absence of eIF2α phosphorylation was correlated, therefore, with earlier expression of myogenin and with the loss of many cells probably as a result of cell death. Indeed, FACS analysis revealed massive cell death of eIF2αS51A expressing myoblasts but not of wild type myoblasts (Figure S2).

### CHOP inhibits myoblast differentiation

**Figure 3:**
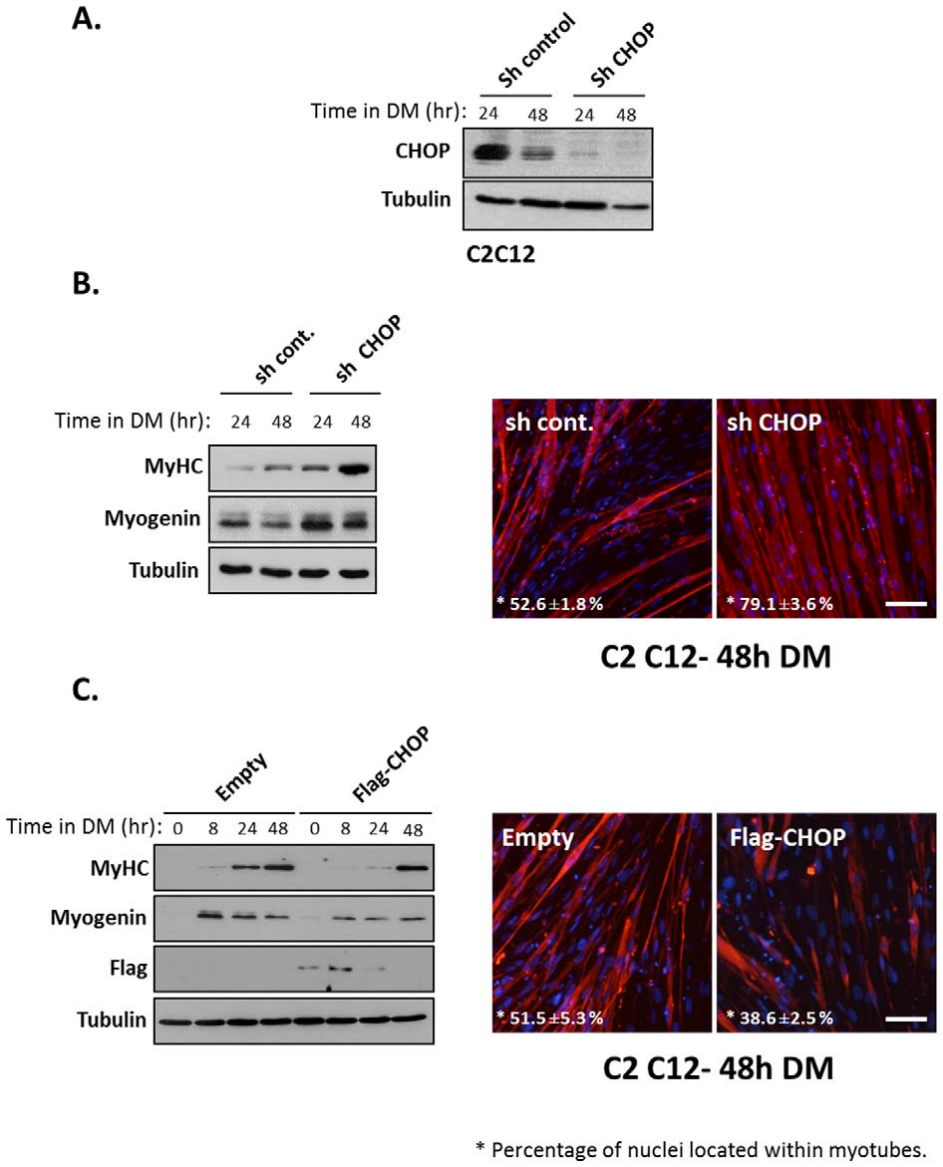
CHOP inhibits myogenic differentiation. (A) CHOP was knockdown in C2C12 myoblasts by infection of lentivirus expressing ShRNA. The levels of CHOP protein were analyzed by Western blot of infected myoblasts. (B) Infected myoblasts were grown in DM for the indicated time periods and myogenic markers were analyzed by Western blot (left panel). Infected myoblasts were grown in DM for 48 hours before cells were immunostained with anti MyHC antibodies (MF20) (right panel) MyHC in red, DAPI in blue. Percentage of nuclei in myotubes was calculated from three independent experiments. Mean values and standard errors are presented. Bar, 50 µm. (C) C2C12 myoblasts were infected with retroviruses encoding a flag-tagged CHOP protein or the parental retrovirus serving as a control. Infected myoblasts were grown in DM for the indicated time periods and myogenic markers were analyzed by Western blot (left panel). Infected myoblasts were grown in DM for 48 hours before cells were immunostained with anti MyHC antibodies (MF20) (right panel) MyHC in red, DAPI in blue. Percentage of nuclei in myotubes was calculated from three independent experiments. Mean values and standard errors are presented. Bar, 50 µm.

To investigate the role of CHOP in myoblast differentiation, its expression was knocked down (Figure 3). As seen in Figure 3A, CHOP protein levels were significantly reduced in the cells expressing shRNA. Loss of CHOP expression had two major outcomes; the expression of myogenin and MyHC were induced earlier and reached higher levels than their levels in control cells (Figure 3B, left panel), and the number of nuclei within differentiated myotubes was significantly increased relative to wild type cells (Figure 3B, right panel). This result suggested that CHOP expression inhibited differentiation of myoblasts to multinucleated myotubes. In a complementary experiment, C2C12 cells were infected with a retrovirus expressing the wild type flag-tagged CHOP protein. Exogenous expression of CHOP delayed and reduced levels of myogenin and MyHC proteins (Figure 3C, left panel). Exogenous CHOP also reduced the number of nuclei within myotubes (Figure 3C, right panel). Interestingly, the levels of the exogenous Flag-CHOP protein diminished with increasing growth period in DM. Overall these results indicated that the transient expression of CHOP delayed myoblasts differentiation.

### Expression of CHOP or MyoD and myogenin are mutually exclusive

**Figure 4:**
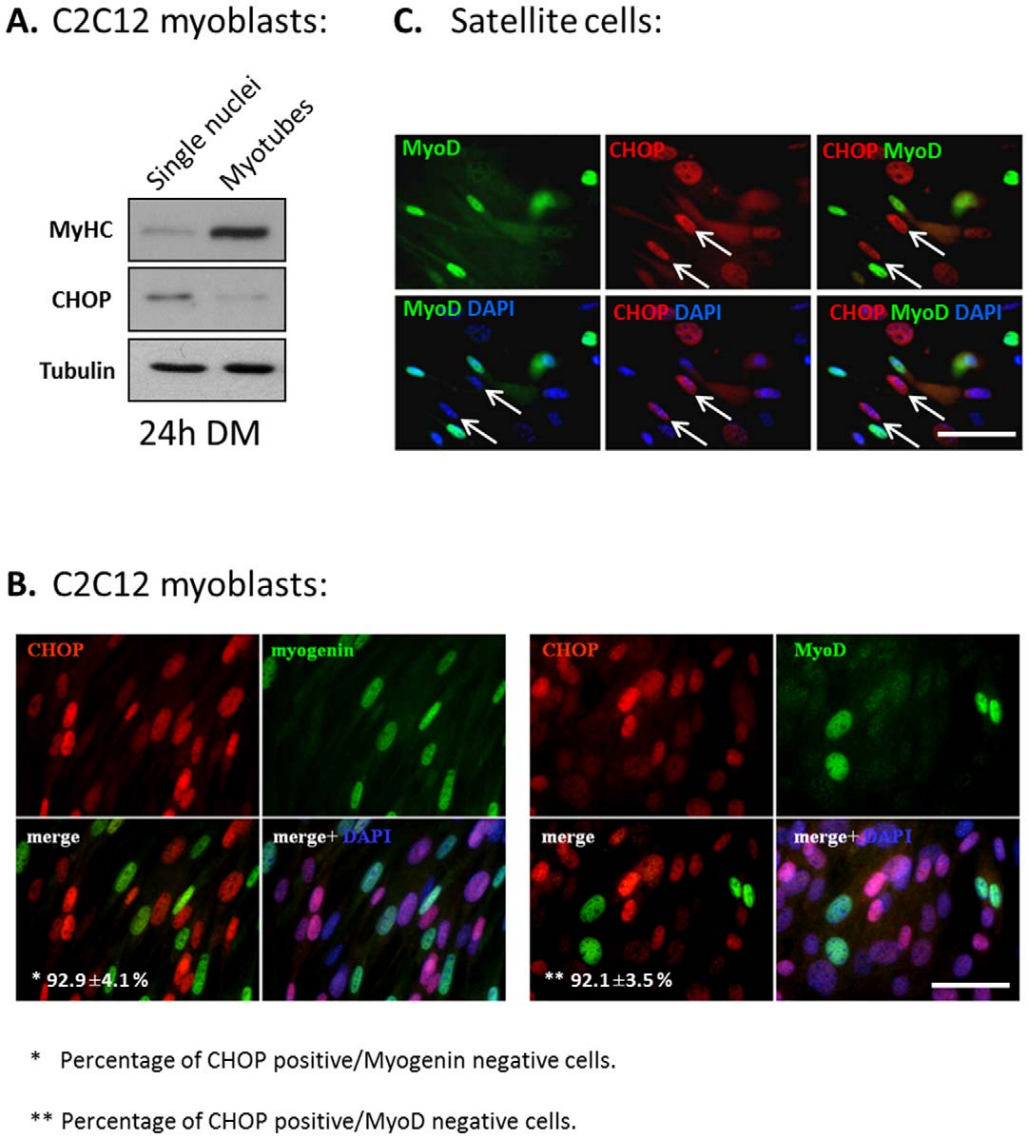
The expression of CHOP and MRFs is mutual exclusive. (A) C2C12 cells were grown in DM for 24 hours and mononucleated cells were separated from myotubes by selective trypsinization. The two cell populations were subjected to Western blot for analyzing the expression of CHOP. (B) C2C12 myoblasts were grown in DM for 24 hours, and double stained with antibodies directed against CHOP and myogenin (left panel) or with antibodies directed against CHOP and MyoD (right panel). DAPI in blue, MyoD and myogenin in green and CHOP in red. Percentage of CHOP positive, myogenin negative and CHOP positive, MyoD negative relative to the total number of CHOP positive cells was calculated in three independent experiments. Mean values and standard errors are presented. Bar, 50 µm. (C) The expression of CHOP in primary satellite cells. To induce their differentiation, satellite cells were grown for 24 hours in GM medium. Cells were analyzed by immunostaining with anti-MyoD (green) and anti-CHOP (red) antibodies. DAPI staining is in blue. Arrows point at nuclei positive for CHOP staining and negative for MyoD staining. Bar, 50 µm.

C2C12 cells growing for 24 hours in DM were divided into two populations; mononuclear cells and multinuclear myotubes [4]. CHOP was mostly expressed in the mononucleated cells that were negative for MyHC expression, and was barely identified in myotubes expressing MyHC (Figure 4A). The presence of a faint band representing CHOP in the myotube fraction was likely the result of residual contamination by mononucleated cells. To monitor the interrelationship between levels of CHOP expression and myogenic regulatory factors, C2C12 cells grown for 24 hours in DM were immunostained for CHOP and MyoD or for CHOP and myogenin (Figure 4B). Interestingly, CHOP was expressed in cells that did not express MyoD or myogenin. To inquire whether CHOP expression was not restricted to only established-myoblast cell line (i.e., C2C12), we followed its expression in primary satellite cells (Figure 4C). More than 95% of the isolated primary cells were MyoD positive under growth conditions, indicating a highly enriched myogenic population (data not shown). When satellite cells were grown in DM, some expressed nuclear CHOP only while others expressed MyoD but not CHOP (Figure 4C). Expression of CHOP or MyoD is thus mutually exclusive in both established myoblasts and primary satellite cells. To investigate whether CHOP was involved in the cell cycle, its expression and the expression of cell cycle proteins were analyzed in C2C12 cells (Figure S3). CHOP expression was not correlated with the expression of cell cycle proteins (Figure S3A), and its ectopic expression did not induce the expression of cyclin-dependent kinase inhibitor, p21 (Figure S3B). Therefore, cells expressing CHOP are not necessarily quiescent as is expected from “reserve cells”.

### An obligatory CHOP repressor prevents the expression of MyoD

**Figure 5:**
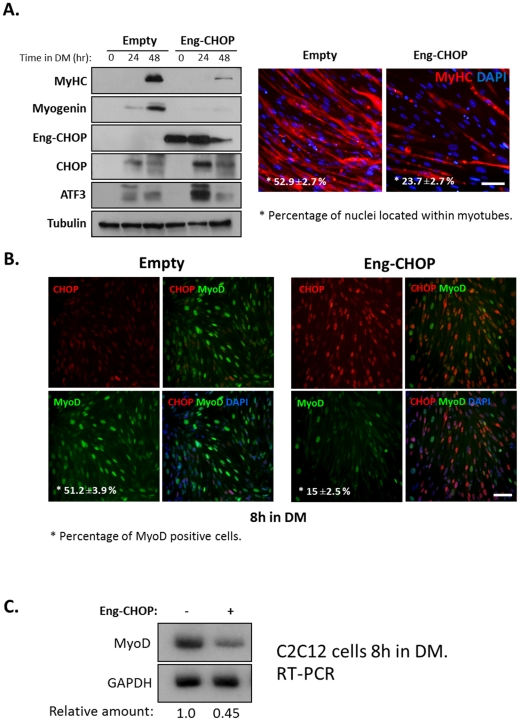
CHOP functions a transcription repressor in myoblasts. (A) A retrovirus encoding a chimera Engrailed-CHOP protein or a retrovirus containing the parental vector was used to infect C2C12 myoblasts. Infected cells were grown in GM and in DM for the indicated time periods and proteins were analyzed by Western blot (left panel). Infected myoblasts were grown in DM for 48 hours and were immunostained with an anti-MyHC antibody (MF20) (right panel). MyHC staining is in red and DAPI is in blue. Percentage of nuclei in myotubes was calculated from three independent experiments. Mean values and standard errors are presented. Bar, 50 µm. (B) Infected cells described in A were grown in DM for 8 hours and were analyzed by immunostaining with antibodies directed against CHOP and MyoD. Control infected cells (left panel) and Engrailed-CHOP infected cells (right panel). Percentage of MyoD-positive nuclei relative to the total number of nuclei was calculated in three independent experiments. Mean values and standard errors are presented. Bar, 50 µm. (C) C2C12 infected cells as in A were grown in DM for 8 hours and total RNA was then extracted. MyoD mRNA levels were analyzed by semi-quantitative RT-PCR and quantified by the posphoimager.

CHOP is a transcription factor that heterodimerizes with basic-leucine zipper transcription factors: Whereas when paired with some it represses transcription, its dimerization with others activates transcription [20], [21], [22], [23]. To determine whether CHOP functions as a transcription activator or a repressor in affecting differentiating myoblasts, we employed two CHOP chimera proteins; VP16:CHOP, an obligatory activator and Engrailed: CHOP, an obligatory repressor. Following confirmation of the expression of the two chimera proteins (Figure S4), we found that ectopic expression of VP16:CHOP in myoblasts did not significantly affect differentiation (data not shown). However, the expression of Engrailed:CHOP profoundly inhibited myogenic differentiation (Figure 5A). Expression of the differentiation markers myogenin and MyHC was almost completely inhibited (Figure 5A, left panel) and the number of nuclei within myotubes was significantly reduced (Figure 5A, right panel). Interestingly, ectopic Engrailed:CHOP chimera mildly increased the levels of endogenous CHOP and ATF3 proteins. Next, Engrailed:CHOP was expressed in myoblasts grown for 8 hours in DM (Figure 5B). In control infected cells, background levels of endogenous nuclear CHOP with significant levels of nuclear MyoD staining were observed (Figure 5B, left panel). In contrast, MyoD staining was absent in cells expressing high levels of CHOP (i.e., Engrailed:CHOP) (Figure 5B, right panel). To determine whether the expression of Engrailed-CHOP affected transcript levels of MyoD, RNA was isolated from control myoblasts and from myoblasts expressing Engrailed-CHOP following their 8 hours growth in DM. Levels of MyoD mRNA were reduced by more than half in cells expressing Engrailed:CHOP (Figure 5C). These results were in line with the proposition that by functioning as a transcriptional repressor in myoblasts CHOP repressed the transcription of MyoD.

### CHOP represses the transcription of MyoD

**Figure 6:**
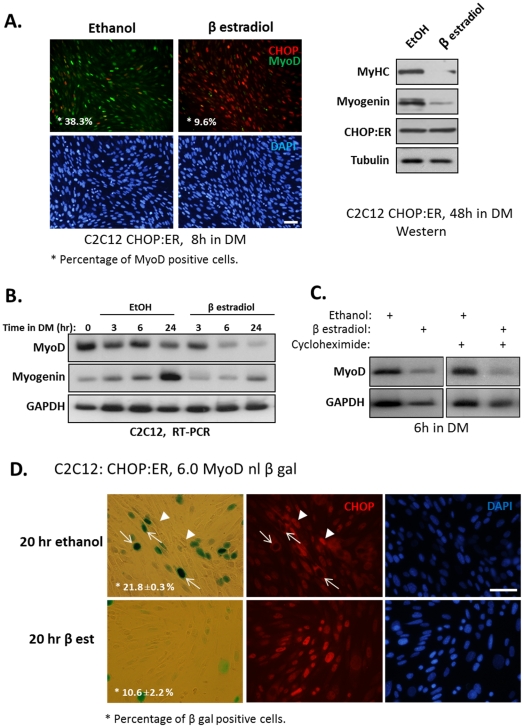
CHOP represses MyoD transcription. (A) A C2C12 derived cell line expressing a chimera CHOP:ER protein was constructed as is described under “Materials and Methods”. (A) Myoblasts were grown in the presence of ethanol or β estradiol (0.1 µM) for 8 hours. Cells were immunostained using anti-MyoD and anti-CHOP antibodies. DAPI in blue, MyoD in green and CHOP in red. Percentage of MyoD-positive nuclei relative to the total number of nuclei was calculated. Bar, 50 µm. (left panel). In another experiment, cells were grown in the presence of ethanol or β estradiol (0.1 µM) for 48 hours and proteins were analyzed by Western blot (right panel). (B) The same cells as above were grown in DM in the presence of ethanol or β estradiol (0.1 µM) for the indicated time periods and mRNA levels of *myod* and *myogenin* were determined by semi-quantitative RT-PCR analysis. (C) The same cells as above were grown in DM and in the presence of ethanol or β estradiol for 6 hours in the absence or presence of cycloheximide added to cells one hour before the addition of ethanol or β estradiol. (D) A clone of the above cells (i.e., expressing CHOP:ER) with integrated MyoD reporter gene (6.0 MyoD -nl β Gal) was isolated. These cells were grown in the presence of ethanol or β estradiol for 20 hours. Nuclear expression of β Gal was identified by an enzymatic colorimetric assay, and the expression of CHOP by immunostaining. Arrows point at β Gal-positive nuclei that are CHOP negative. Percentage of β Gal-positive nuclei out of the total number of nuclei was calculated in two independent experiments. Mean values and standard errors are presented. Bar, 50 µm.

To further inquire how CHOP lowered MyoD transcript levels, we employed a chimera protein of CHOP and the hormone binding site of estrogen receptor (CHOP:ER). Following addition of β estradiol to the cell medium, the cytoplasmic CHOP:ER protein was translocated into the nucleus (data not shown). Importantly, CHOP:ER chimera inhibited differentiation of C2C12 cells that were grown in the presence of β estradiol as was apparent by the reduced expression of myogenin and MyHC relative to their levels in the same cells that were grown in the presence of ethanol (Figure 6A, right panel). Immunostaining indicated that translocation of CHOP:ER to cell nuclei following the addition of β estradiol, largely inhibited the expression of MyoD (Fig 6A, left panel). Next, we asked how the activation of CHOP:ER chimera affected MyoD and myogenin mRNA levels (Figure 6B). The level of *myod* mRNA was significantly lowered after 6 hours of growth in the presence of β estradiol relative to control cells grown for the same period of time in the presence of ethanol. The level of *myogenin* mRNA that was significantly elevated following 24 hours of growth in DM and ethanol remained low when the same cell line was grown for 24 hours in DM and β estradiol. This result strongly indicated that temporal activation of CHOP lowered *myod* transcript levels and prevented the subsequent increase in *myogenin* mRNA levels. Moreover, when β estradiol was replaced after several hours by ethanol, levels of MyoD mRNA were restored to the levels that were attained before CHOP activation (data not shown). Therefore, CHOP-mediated lowering of the level of MyoD mRNA was reversible. To determine whether the decrease in MyoD transcripts by CHOP required newly synthesized proteins, cycloheximide was added during the activation of CHOP:ER (i.e., addition of β estradiol). Levels of MyoD transcripts were similarly reduced after the activation of CHOP in the absence or in the presence of cycloheximide (Figure 6C). Therefore, protein synthesis was not required for CHOP-mediated lowering of MyoD mRNA levels raising the likely possibility that CHOP directly repressed MyoD transcription.

To inquire whether CHOP affected MyoD expression by interacting directly with transcription regulatory sequences of MyoD, we used a reporter gene containing promoter and enhancer sequences of MyoD (6.0 MyoD- nl β gal) that was stably integrated into the genome of chimera CHOP:ER-expressing C2C12 myoblasts [24]. A clone of cells expressing β galactosidase in 40–50% of the cell nuclei of growing myoblasts (in GM) was isolated and further analyzed. This cell line was grown in DM for 20 hours in the presence of ethanol (inactive CHOP) or β estradiol (active CHOP). Nuclear β gal was detected by colorimetric assay, while CHOP expression was monitored by immunofluorescent staining (Figure 6D). Our results show that the number of CHOP-positive nuclei was significantly increased while the number of β gal positive nuclei decreased in β estradiol-treated myoblasts relative to control myoblasts that were treated with ethanol (Figure 6D). Interestingly, the most intense β gal staining occurred in cells expressing cytoplasmic CHOP (upper panel, arrows) while cells expressing nuclear CHOP (upper panel, arrowheads) were negative for β gal staining. This result indicated that nuclear CHOP repressed expression driven by transcription regulatory sequences of the *myod* gene.

### CHOP binds to transcription regulatory sequences of *myod* and affects histone acetylation

**Figure 7:**
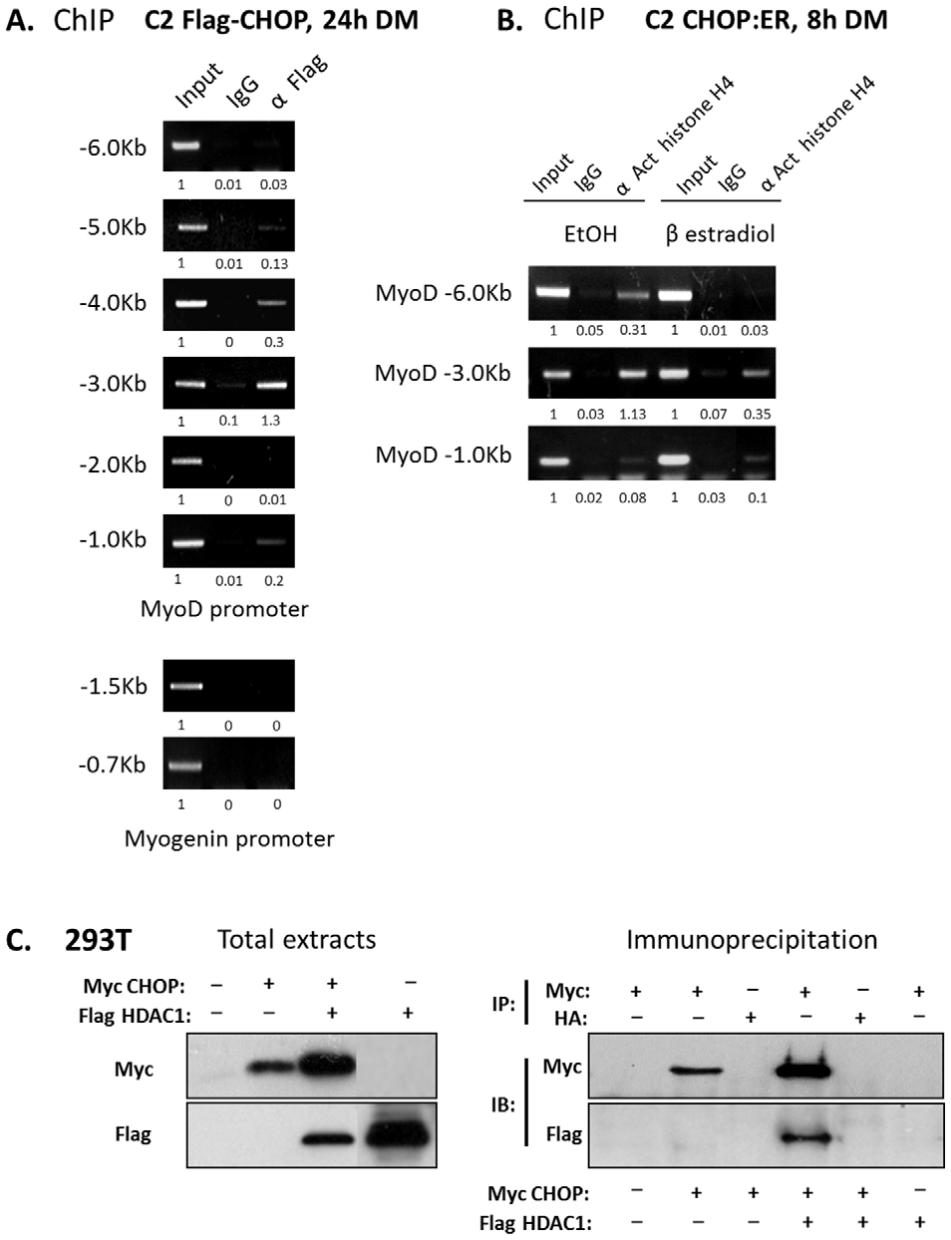
CHOP associates with MyoD regulatory sequences and affects histone acetylation. (A) A Chromatin IP experiment was performed on C2C12 cells expressing Flag-CHOP that were grown in DM for 24 hours. Immunoprecipitation of fragmented DNA was performed with anti-Flag antibodies. PCR amplification of fragments scattered throughout 6 Kb upstream region of the MyoD transcription unit and along 2 Kb upstream of the myogenin transcription unit was performed. PCR fragments were separated over agarose gels. Gels were scanned and values of band intensities are presented below. For each set of PCR primers, the value of the input was set to 1. (B) C2C12 cells expressing CHOP:ER chimera were grown in DM for 8 hours in the presence of ethanol or β estradiol (0.1 µM). Chromatin IP assay was performed on fragmented DNA with anti-acetylated histone H4 antibody. PCR amplification of fragments scattered along 6 Kb upstream of the MyoD transcription unit was performed. Gels were scanned and values of band intensities are presented below. For each set of PCR primers, the value of the input was set to 1 (C) 293T cells were transfected with expression plasmids as indicated. Cells were lysed under mild conditions and extracted proteins were analyzed (left) or immunoprecipitated with anti-Myc or anti-HA epitope antibodies as indicated (right). Proteins were analyzed by Western blotting as indicated.

We next explored the possibility that CHOP affected *myod* transcription by associating with its upstream transcription regulatory sequences. Chromatin Immunoprecipitation (ChIP) analysis was performed in C2C12 myoblasts stably expressing Flag-CHOP and grown in DM for 24 hours. The use of ectopically expressed tagged-CHOP was necessary since the commercial anti CHOP antibody (9C8) did not immunoprecipitate detectable levels of endogenous CHOP protein from myoblasts grown in DM for 24 hours (data not shown). Therefore, levels of endogenous CHOP protein are likely to be too low for detection by IP. The regulatory sequences controlling *myod* transcription in myoblast cell lines and in primary satellite cells are located within 6 kb upstream to the transcription initiation site [24]. Chromain IP of Flag-CHOP followed by PCR analysis of fragments that were scattered throughout *myod* and *myogenin* upstream sequences was performed (Figure 7A). Association of CHOP with several regions of *myod* upstream sequences, most prominently around -3Kb was observed. By contrast, association of CHOP with *myogenin* upstream sequences could not be detected. Therefore, we conclude that CHOP interacts with *myod* upstream sequences and through this interaction it might repress *myod* transcription. To investigate the possible involvement of CHOP with the chromatin of *myod* regulatory sequences, localized histone modifications were investigated in the CHOP:ER expressing myoblast cell line. This cell line was selected for this analysis since it enables a comparison of nuclear active CHOP with cytoplasmic inactive CHOP (+/− β estradiol) under uniform growth conditions and in the absence of endogenous CHOP expression (8 hours in DM). Chromatin IP of acetylated histone H4 (“activated chromatin”) followed by PCR analysis of fragments that were scattered throughout *myod* upstream sequences was performed. Histon H4 acetylation was identified in several regions upstream to the initiation site of the transcriptionally-active promoter (Figure 7B) (+ethanol; inactive CHOP:ER). Following the activation of CHOP:ER (+β estradiol), histone acetylation was significantly reduced around the 6kb upstream region and less significantly around the 3Kb upstream region. The 6Kb upstream region includes the distal regulatory region (DRR) containing *myod* enhancer sequences [24]. It is likely, therefore, that nuclear CHOP protein recruits histone deacetylase to the upstream regulatory sequences of the *myod* gene. To investigate the possibility that CHOP interacts with histone deacetylase (HDAC), 293T cells were transfected with expression vectors of epitope-tagged CHOP, HDAC1, HDAC3 and HDAC4. CHOP was immunoprecipitated from cells under mild detergent conditions and presence or absence of the different HDACs in the protein complex was assessed (Figure 7C). Interestingly, HDAC1 was found to be associated with CHOP (Figure 7C, right panel) whereas HDAC3 and 4 were not detectable in the CHOP-containing complexes (data not shown). This result is in line with the idea that histone tail deacetylation by CHOP at MyoD regulatory sequences involves recruitment of HDAC1. To investigate the possible involvement of HDACs in MyoD expression, an HDAC inhibitor, trichostatin A (TSA) was added to differentiating C2C12 cells (Figure S5). The number of nuclei within differentiated myotubes was significantly increased in TSA-treated cells relative to wild type cells. Moreover, unlike control cells, a significant number of TSA-treated cells co-expressed CHOP and MyoD, indicating that HDAC inhibitors may have prevented CHOP-mediated repression of MyoD expression.

## Discussion

### Temporal activation of stress-induced genes during myoblast differentiation

Phosphorylation of eIF2α occurring in response to diverse stress conditions, reduces global translation and allows cells to overcome the damage and recuperate [25]. The transient phosphorylation of eIF2α during the *in vitro* differentiation of myoblasts as observed in the present study is indicative of stress occurring during this process. Indeed, our results indicate that this phosphorylation event is accompanied by transient attenuation of protein synthesis. Phosphorylated eIF2α is unable to initiate translation from most mRNA molecules, but selectively translate specific mRNA species such as mRNA for the transcriptional activator ATF4 [26], [27], [28]. In this study we were unable to detect ATF4 protein probably due to technical problems, however we did detect the expression of its two targets, ATF3 and CHOP [20], [29]. The expression of CHOP and ATF3 occurred in myoblasts and not in fibroblasts grown under conditions of serum starvation indicating that this response is integral to the myogenic process. Morishima and colleagues [17] came to the conclusion that ER stress occurring during myoblast differentiation induced UPR response transmitted by ATF6. They showed that a pathway downstream to ATF6 that involves the activation of caspase12 induced apoptosis of a subset of cells during myoblast differentiation. Dynlacht and colleagues [10], [11] identified ATF4 and XBP1as probable direct targets of MyoD and suggested that muscle exhibited properties of an active UPR under physiological conditions. They proposed the existence of “ER stress control checkpoint” regulated by the levels of spliced XPB1, that would halt the differentiation process until ER homeostasis is reached [11]. Indeed, expression of spliced XBP1 reduced myotube formation. Together, these studies suggest that all three UPR sub-pathways are being activated during myoblast differentiation and myofiber maintenance. Activation of each pathway may exhibit different response to stress; whereas activated ATF6 induces apoptotic cell death of some cells, the induction of XBP1and of eIF2α-CHOP delays the differentiation process in others. We show that blockage of the eIF2α-CHOP pathway in myoblasts derived from eIF2αS51A knockin mouse underwent massive cell death during the differentiation process. The result indicates that this pathway may function as a stress control checkpoint necessary to delay the differentiation process before damage has been repaired [10], [11].

### CHOP temporarily delays the differentiation process

The results presented here indicate that the expression of CHOP delays the differentiation of myoblasts. CHOP is only expressed in mononucleated undifferentiated cells. At any time point analyzed by immunostaining, CHOP was detected in a subset of cells (usually between 40–80% of the cells). By the type of experiments performed, we cannot resolve whether over time CHOP is transiently expressed in all or only in a sub-set of cells. Since the differentiation process is not synchronized in culture we may assume that CHOP is probably expressed in all myoblasts at different times. Every cell expressing CHOP did not express the myogenic regulatory factors, MyoD and myogenin. Therefore, since CHOP is expressed in a clonal myogenic culture in which all cells express MyoD under proliferation conditions, we concluded that upon induction, CHOP represses myogenic gene expression. The downregulation of MyoD expression in a subset of myoblasts during *in vitro* differentiation was reported before and these cells were termed “reserve cells” [30]. The expression of MyoD was transiently lost in myoblasts grown in conditions of poor serum, and was regained when the same cells returned to growth conditions of high serum. In the present study we used an inducible chimera CHOP protein and could demonstrate that the expression of MyoD was regained after the inactivation of CHOP. Therefore CHOP may play a role in the plasticity of myoblasts to temporarily delay differentiation and restore more “stem-like” properties. Animal studies should reveal whether CHOP expression functions as a necessary checkpoint in the differentiation of embryonic as well as adult myogenic lineage.

### CHOP is a repressor of MyoD transcription

CHOP heterodimerizes with other bZIP proteins like C/EBPs, AP1, CREB, ATF3 and ATF4 [20] and different heterodimers may activate or repress transcription [21], [31], [32], [33]. Our finding that the expression of Engrailed-CHOP in myoblasts mimicked wild type CHOP in inhibiting MyoD expression indicates that CHOP functioned as a repressor of MyoD. Yet, since endogenous CHOP was mildly induced by the expression of Engrailed-CHOP, we cannot exclude the possible involvement of the endogenous CHOP as an activator. Therefore, in myoblasts, CHOP may activate the transcription of some genes while repressing the transcription of others. Several experiments indicate that CHOP functions as a direct transcriptional repressor of the *myod* gene. First, CHOP affects the transcript levels of *myod*. Expression of Engrailed-CHOP decreased *myod* transcript levels (Figure 5C), while knockdown of CHOP entails an increase in the level of *myod* transcripts (J.A. unpublished results). Second, an inducible CHOP protein, CHOP:ER, decreased the levels of *myod* transcripts upon its translocation into cell nuclei. Thus, only nuclear CHOP can affect *myod* transcript levels. Third, nuclear CHOP:ER reduced the level of *myod* mRNA in the presence of the translation inhibitor, cycloheximide, indicating that CHOP exerted this effect without the involvement of newly synthesized mediators. Forth, a genome-integrated *myod* promoter-enhancer reporter gene was expressed in myoblasts expressing cytoplasmic transcriptionally inert CHOP:ER protein but was not expressed in cells expressing a nuclear and functional CHOP:ER protein. Therefore, nuclear CHOP affected MyoD expression via the upstream regulatory sequences of the gene. A recent study showed that heterodimers of CHOP with ATF4 repress transcription of *aspargine synthetase* gene by association with its transcription regulatory sequences [33]. Indeed, ChIP assay performed in the present study revealed that CHOP associated with a certain region of *myod* regulatory sequences mostly around 3.0 Kb upstream of the transcriptional initiation site. By contrast, CHOP was not associated with *myogenin* regulatory sequences, indicating that repression of *myogenin* transcription by CHOP occurred indirectly, probably through the direct inhibition of MyoD transcription. Presently, we did not identify the exact CHOP binding sites at *myod* regulatory sequences. Since, the dimerization domain of CHOP is also necessary for its function (J.A. unpublished results) it is plausible that CHOP heterodimers repress *myod* transcription. Identifying the heterodimeric partner of CHOP should facilitate the search for putative binding sites at the regulatory sequences. In that respect, association of C/EBP with a CArG element in the MyoD DRR was described before [34]. However, binding of CHOP to this region was not identified by ChIP analysis in the present study.

### CHOP deacetylates chromatin at upstream regulatory sequences of the *myod* gene

The regulatory sequences necessary for MyoD expression in satellite cells are included within a 6Kb DNA fragment upstream to the transcription initiation site [24]. Two regions within these sequences are sufficient for the expression of MyoD; the proximal regulatory region (PRR, 275 bp promoter sequences) and the distal regulatory region (DRR, 750 bp enhancer sequences located around 6Kb upstream sequences) [24], [34], [35], [36], [37]. In the present study we find that CHOP associates most significantly with sequences that are located around 3Kb upstream to the transcription initiation site, found in between DRR and PRR. This regulatory region was not identified before, probably because it mediates repression of *myod* transcription, unlike activation that is mediated by DRR and PRR. We also find that CHOP is active in the deacetylation of histone H4 at two regulatory regions; around 6Kb including the DRR and around 3Kb upstream sequences. CHOP activity in histone deacetylation at a distal region (DRR) might be explained by long-range protein-protein interactions and DNA looping [38]. Removal of acetyl moieties form histones is one of several measures to repress transcription [39]. Our results indicate that CHOP interacts with histone deacetylase1 (HDAC1) in cells, yet further experiments will be necessary to prove this possibility in myoblasts. Therefore, we would like to propose a model in which CHOP recruits HDAC1 to MyoD regulatory sequences and by deacetylation of histone tail counteracts the sustained activity of enhancer bound (DRR) histone acetylases [40]. Once, the expression of CHOP is terminated, *myod* default transcription is regained. This mechanism may explain the transient nature of MyoD repression. Interestingly, CHOP activity did not affect K27 histone trimethylation at several loci, a signature of Polycomb-mediated repressed heterochromatin (J.A. unpublished result). This result further indicates that the plasticity of CHOP-mediated repression is achieved by reducing histone acetylation rather than inducing a more stable heterochromatic repressive chromatin.

## Materials and Methods

### Cell culture and satellite cell isolation

C2C12 cells were a gift from Dr. David Yaffe [41]. Cell lines were maintained in Dulbecco's modified Eagle's medium supplemented with 15% calf serum (HyClone), penicillin, and streptomycin (growth medium, GM). To induce differentiation, we used Dulbecco's modified Eagle's medium supplemented with 10 µg of insulin per ml and 10 µg of transferrin per ml (differentiation medium, DM). Fibroblasts from different mice strains (3T3 wild type, 3T3 eIF2αS51A knockin) were infected with a retrovirus encoding the MyoD protein and the hormone binding domain of estrogen receptor (pBABE puro MyoD:ER) [18]. Myoblast cell lines were isolated following selection with puromycin (3 µg/ml). Addition of 10^−7^M β-estradiol to DM induced translocation of the cytoplasmic chimera protein into the nucleus and initiation of the myogenic program. Satellite cells were isolated from the hind legs of 3- to 4-week-old mice. Animal experiments performed in this study were specifically approved by the Technion Committee for Care and Use of Laboratory Animals (IL-020-02-2010; valid until February 2014). The Technion holds a valid assurance (#A5026-01) of the US Department of Health and Human Services for humane care and use of laboratory animals. Muscle tissues were separated from bones and cartilage dissected and minced, followed by enzymatic dissociation at 37°C with 0.25% trypsin-EDTA for 30 min. Cells were filtered through 100 µm membrane (Cell strainer, BD Falcon) and were cultured in rich proliferation medium (BIO-AMF-2, Biological Industries, Ltd.). To isolate satellite cells from fibroblasts, a preplating technique was employed [42], which separates myogenic cells based on their adherence to gelatin-coated flasks. To induce differentiation, cells were grown in DMEM containing 5% horse serum (Biological Industries) for up to 4 days.

### Retroviral expression vectors and infections

pBABE puro CHOP was described before [43], [44]. pCLNCX-Flag-CHOP: A PCR fragment was isolated from pBABE puro-CHOP vector and cloned into pCLNCX v.2 using HindIII-ClaI linkers. pCLNCX-Eng-CHOP: A PCR fragment of CHOP was inserted into pCS2+ ENG-N vector using XhoI-XbaI linkers. Eng-CHOP reading frame was PCR isolated and was inserted into pCLNCX v.2 vector using HindIII-ClaI linkers. pCLNCX-VP16-CHOP: A PCR fragment of CHOP was inserted into pCS2+ VP16-N vector using XhoI-XbaI linkers. VP16-CHOP was PCR isolated from pCS2+ VP16-CHOP and was inserted into pCLNCX v.2 vector using HindIII-ClaI linkers. pBABE puro-CHOP:ER: CHOP fragment was inserted into pBabepuro:hbER vector using BamHI-EcoRI linkers. Infection of myoblasts with replication-defective retroviruses was used to generate cell lines expressing the different CHOP proteins. Retroviruses were generated by transfection of retroviral vectors and an expression vector of vesicular stomatitis virus, the glycoprotein (VSVG), into viral packaging cells, 293gp, expressing the *gag* and *pol* genes [45].The medium of transfected 293gp cells containing retroviruses was used to infect cells. Forty-eight hours later, infected cells were used for the specific experiments. In all cases, infection efficiency was higher than 80%.

### ShRNA-mediated knockdown of proteins

Knockdown of the CHOP protein in myoblasts was achieved by lentiviral infections of 5 viral vectors that express different shRNA directed to CHOP mRNA and were purchased from Sigma-Aldrich (ShRNA MISSION). Viruses were generated by transfection of 293T cells with MISSION shRNA vectors and ΔNRF vector encoding for gag- pol, and CMV-VSVG encoding for envelop glycoprotein of vesicular stomatitis virus. The medium of transfected 293T cells containing lentiviruses was used to infect myoblasts that were further selected with puromycin (3 µg/ml). Knockdown efficiency was analyzed by western blotting. Viral particles that caused maximal repression of CHOP expression relative to control particles were chosen for the knockdown experiments.

### Western blotting

The procedure was performed as described [46]. The following antibodies were used in the immunoblatting: anti CHOP monoclonal (9C8); 1∶100, anti ATF3 (Santa cruz);1∶500, anti eIF2α (phospho S51, Cell signaling); 1∶1000, anti MyoD (Santa Cruz #760); 1∶1000, anti myogenin, (F5D); 1∶1000, anti MyHC (MF20); 1∶1000, anti Flag (M2, Sigma-Aldrich); 1∶1000, anti α tubulin (Sigma- Aldrich); 1∶10000. Proteins were visualized using the enhanced chemiluminescence kit of Pierce Inc.

### Immunostaining

Cells were fixed and immunostained as described [47]. The primary antibodies used were anti-MyHC (MF-20), anti-CHOP (9C8), anti MyoD (Santa cruz#760), anti myogenin (F5D), anti-estrogen receptor (Santa cruz). Cells were exposed to secondary fluorescently-labeled antibody and DAPI (1 µg/ml). The immunochemically stained cells were viewed at x200 or x650 magnification under a fluorescence microscope and photographed with a digital camera.

### Semi quantitative RT-PCR analysis of RNA

RNA extraction, reverse transcriptase and PCR reactions were performed as described [46]. The following pairs of primers were used: MyoD: **F;** 5′-AGCACTACAGTGGCGACTCA-3′ **R**; 5′-CTGGGTTCCCTGTTCTGTGT-3′ Myogenin: **F;** 5′-CTACAGGCCTTGCTCAGCTC-3′ **R;** 5′-GGCAACAGACATATCCTCCA-3′ GAPDH: **F;** 5′ ACA TCA TCC CTG CAT CC 3′ **R;** 5′ CTC CTT GGA GGC CAT GT 3′.

### Separation of mononucleated cells from myotubes

In general, myotubes were separated from mononucleated cells by selective trypsinization as is described [4]. Briefly, C2C12 cells that were grown in DM for 24 hours were trypsinized with 0.05% trypsin for 0.5 minute which specifically detached myotubes and leaving undifferentiated mononucleated cells adherent to the dish.

### Chromatin IP analysis

Chromatin immunoprecipitation (IP) assay was performed after Tapscott and colleagues [9]. The cells used for ChIP assays were C2C12 myoblasts expressing Flag-CHOP to identify CHOP binding to DNA and C2C12 myoblasts expressing CHOP:ER to identify histone H4 acetylation. Antibodies (5 µl) used for immunoprecipitations were anti-Flag (Sigma), anti-acetyl H4 antiserum (Upstate), or IgG (Santa cruz). PCR reactions were performed with cold nucleotides and were analyzed over agarose gels. For the input samples, 2 ng DNA was used and 40 cycles were performed. For CHOP IPs and acetylated H4 IPs 1% of the IP sample was used and 40 cycles were performed. The following pairs of primers were used:

MyoD -6 to -5 Kb (-5586) F: GCCCGCAGTAGCAAAGTAAG (-5315) R: ATAGGTGGCCCCTTTGATTT. Size: 271 bp. MyoD -5 to -4Kb (-4488) F: CAGCTGAGGTTCCAAAAAGG (-4362) R: ATGGATGTGGGGTTCATCAT. Size: 126 bp. MyoD -4 to -3Kb (-3327) F: CAGGGTGCTGGTGCTCTTAT (-3121) R: AAACCGGCAGGAATAGGACT Size: 206 bp. MyoD -3 to -2Kb (-2631) F: CTTTGCCCGCATGTAAGTTT (-2367) R: GGCCACTCAGATGGTTGTTT. Size: 264 bp. MyoD -2 to -1Kb (-1534) F: CCTGGGTGAGTCTGATGGAT (-1293) R: TTGTGGGATCTCTGGCTCTC. Size: 241 bp. MyoD -1 Kb to start. (-586) F: AATAGCACTGCCACCGATTC (-407) R: GATTGCAGGAGGTTTGGAGA. Size: 179 bp. Myogenin -1.5 to -0.7Kb (-1487) F: GCCCAGGACAGACAAATGAT (-1251) R: AGCCTCACAAGAGGCAGCTA. Size: 236 bp. Myogenin -0.7 Kb to start. (-442) F: GGATTTTCAAGACCCCTTCC (-221) R: CCGTCGGCTGTAATTTGATT. Size: 221 bp.

(The first nucleotide numbering of each primer is relative to the transcription start site.)

### Co-immunoprecipitation

Proteins were extracted in lysis buffer (20mM Tris pH7.5, 150mM NaCl, 1mM EDTA, 1mM EGTA, 1% Tritin X-100, a cocktail of protease inhibitors). 500 µg of proteins were incubated with 40 µ 50% protein A/G beads for an hour (pre-clearing) and then with protein A/G beads that were pre incubated with the indicated antibodies (5 µg each) for 4 hours. Beads were washes 5 times in lysis buffer (1 ml). Proteins were released from beads in SDS sample buffer and were analyzed by western blotting.

## Supporting Information

Figure S1 **Translational switch during myoblast differentiation.** C2C12 myoblasts were differentiated in DM for the indicated time periods. Two hours before protein extraction, cells were metabolically-labeled with 100 µCi/ml [^35^S] methionine. Identical total amount of proteins of each sample were loaded and were separated over SDS-PAGE. Gel was exposed to X ray films.
(TIF)

Figure S2 **Significant cell death of eIF2αS51A myoblasts**. Mutated eIF2αS51A and wild type fibroblasts were infected with viruses encoding the MyoD:ER protein. Cell lines were grown in DM for the indicated time periods. Cells were fixed and DNA was labeled with propidium iodide, and cells were FACS analyzed. DNA content was quantified using ModFit software (Becton Dickinson).
(TIF)

Figure S3 **CHOP does not induce the expression of p21 CDK inhibitor.** (A) C2C12 cells were differentiated for 24 hours in DM. Upper panel: Cells double-stained with antibodies against CHOP and p21 (Santa Cruz). Arrows point at nuclei positive for CHOP staining and negative for p21 staining. Lower panel: Cells double-stained with antibodies against CHOP and phospho Histone H3 (Cell signaling). Arrow points at CHOP^+^/pHis H3^+^ cell. Bar, 50 µm. (B) Control C2C12 ER and C2C12 CHOP:ER cells were grown for 8 hours and 24 hours in DM and in the presence of ethanol or β estradiol (0.1 µM). Levels of p21 protein were analyzed by Western blotting.
(TIF)

Figure S4 **Expression of CHOP chimera proteins.** 293T cells were transfected with retroviral expression vectors encoding for wt CHOP, VP16-CHOP and Eng-CHOP. Twenty four hours after transfection, cells were lysed and protein extracts were analyzed by Western blot with anti-CHOP antibodies.
(TIF)

Figure S5 **Treatment of C2C12 cells with trichostatin A (TSA) increases co-expression of CHOP and MyoD.** C2C12 cells were grown for 10 hours in DM and in the absence or presence of trichostatin A (50 nM). Following that period, medium was replaced by DM. (A) Cells were grown for additional 38 hours (total 48h in DM) and were then immunostained with antibodies against NyHC. Percentage of nuclei within myotubes was calculated from two microscopic fields. Bar, 50 µm. (B) Cells were grown for additional 14 hours (total 24h in DM) and were immunostained with antibodies against CHOP and MyoD. Percentage of CHOP^+^/MyoD^+^ nuclei was calculated from 100 CHOP positive nuclei. Bar, 50 µm.
(TIF)
