# Supplementary figures and images for: Stress-Induced C/EBP Homology Protein (CHOP) Represses MyoD Transcription to Delay Myoblast Differentiation
Source: PLoS One. 2011 Dec 29;6(12):e29498. doi: 10.1371/journal.pone.0029498 (PMC3248460; doi:10.1371/journal.pone.0029498)

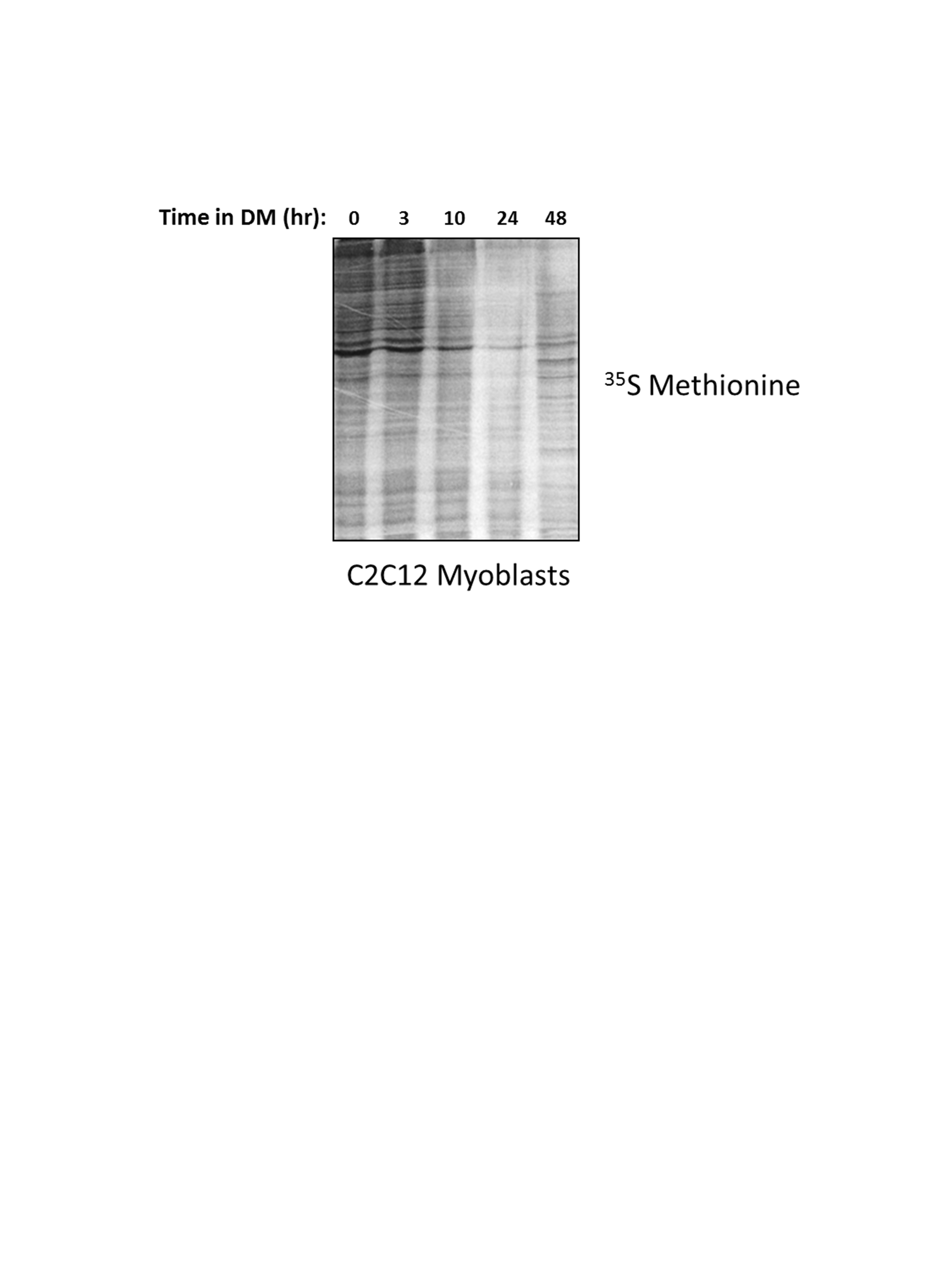

Supplement: Figure S1 — Translational switch during myoblast differentiation. C2C12 myoblasts were differentiated in DM for the indicated time periods. Two hours before protein extraction, cells were metabolically-labeled with 100 µCi/ml [35S] methionine. Identical total amount of proteins of each sample were loaded and were separated over SDS-PAGE. Gel was exposed to X ray films. (TIF) [file pone.0029498.s001.tif]

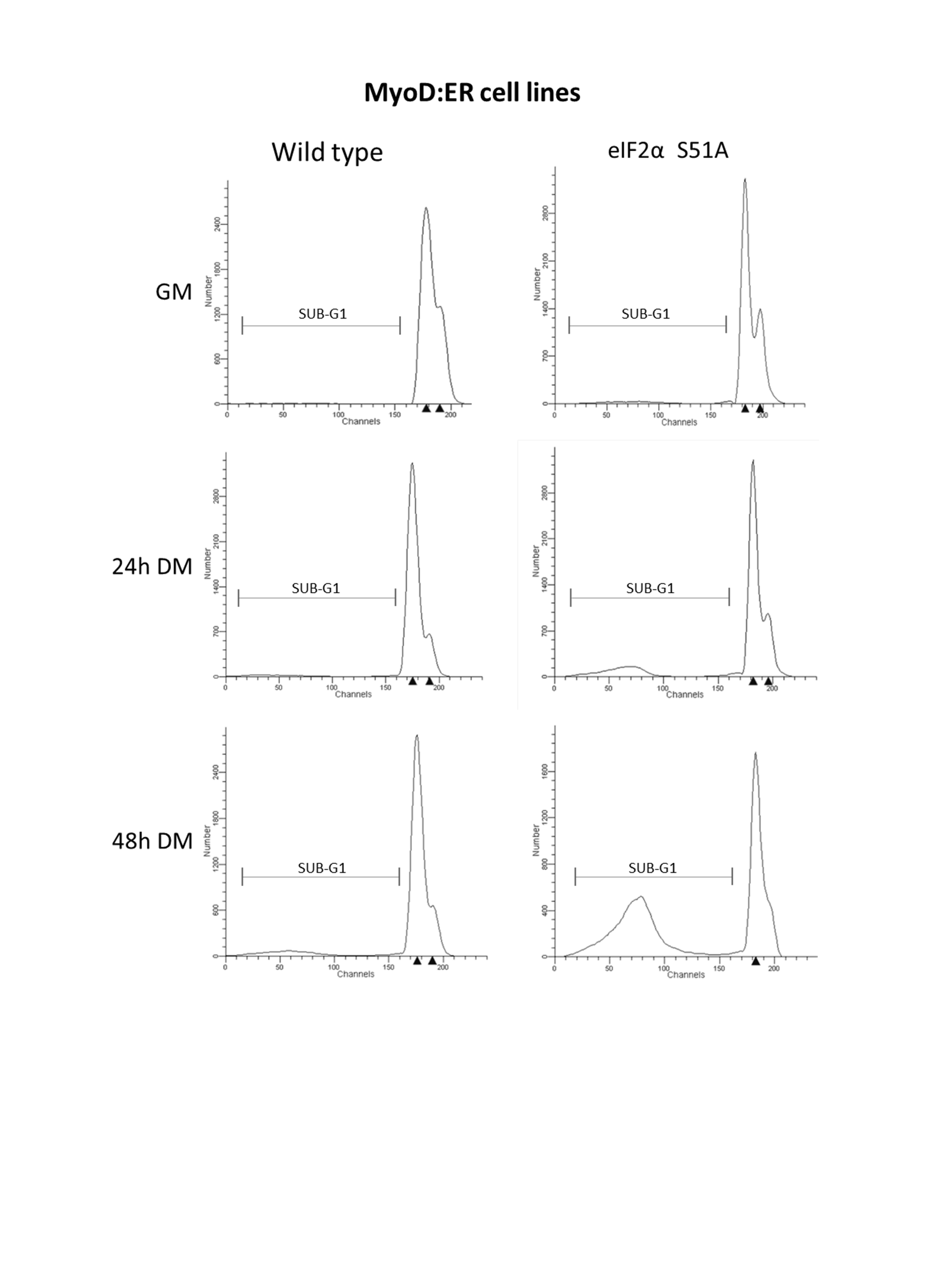

Supplement: Figure S2 — Significant cell death of eIF2αS51A myoblasts. Mutated eIF2αS51A and wild type fibroblasts were infected with viruses encoding the MyoD:ER protein. Cell lines were grown in DM for the indicated time periods. Cells were fixed and DNA was labeled with propidium iodide, and cells were FACS analyzed. DNA content was quantified using ModFit software (Becton Dickinson). (TIF) [file pone.0029498.s002.tif]

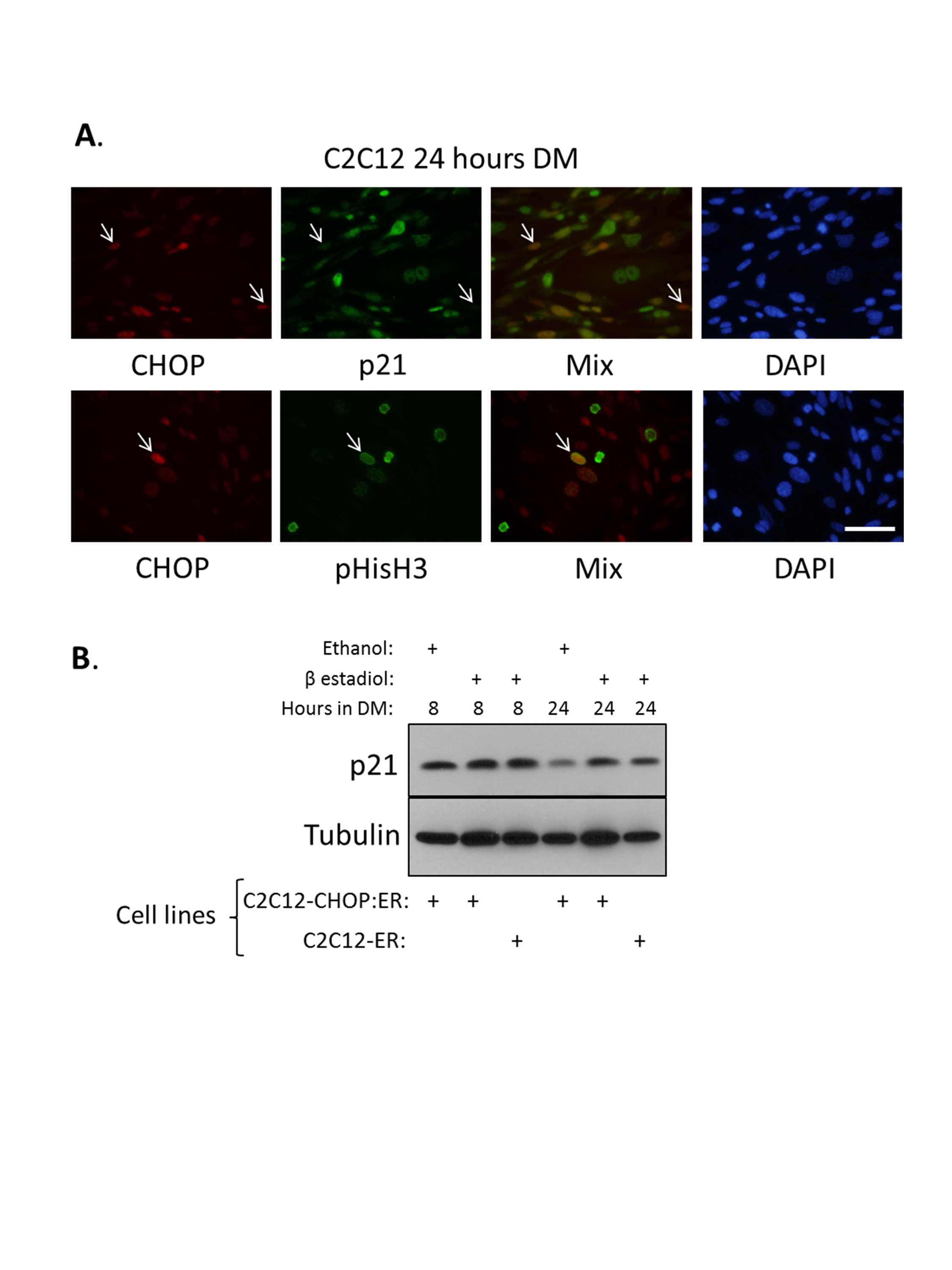

Supplement: Figure S3 — CHOP does not induce the expression of p21 CDK inhibitor. (A) C2C12 cells were differentiated for 24 hours in DM. Upper panel: Cells double-stained with antibodies against CHOP and p21 (Santa Cruz). Arrows point at nuclei positive for CHOP staining and negative for p21 staining. Lower panel: Cells double-stained with antibodies against CHOP and phospho Histone H3 (Cell signaling). Arrow points at CHOP+/pHis H3+ cell. Bar, 50 µm. (B) Control C2C12 ER and C2C12 CHOP:ER cells were grown for 8 hours and 24 hours in DM and in the presence of ethanol or β estradiol (0.1 µM). Levels of p21 protein were analyzed by Western blotting. (TIF) [file pone.0029498.s003.tif]

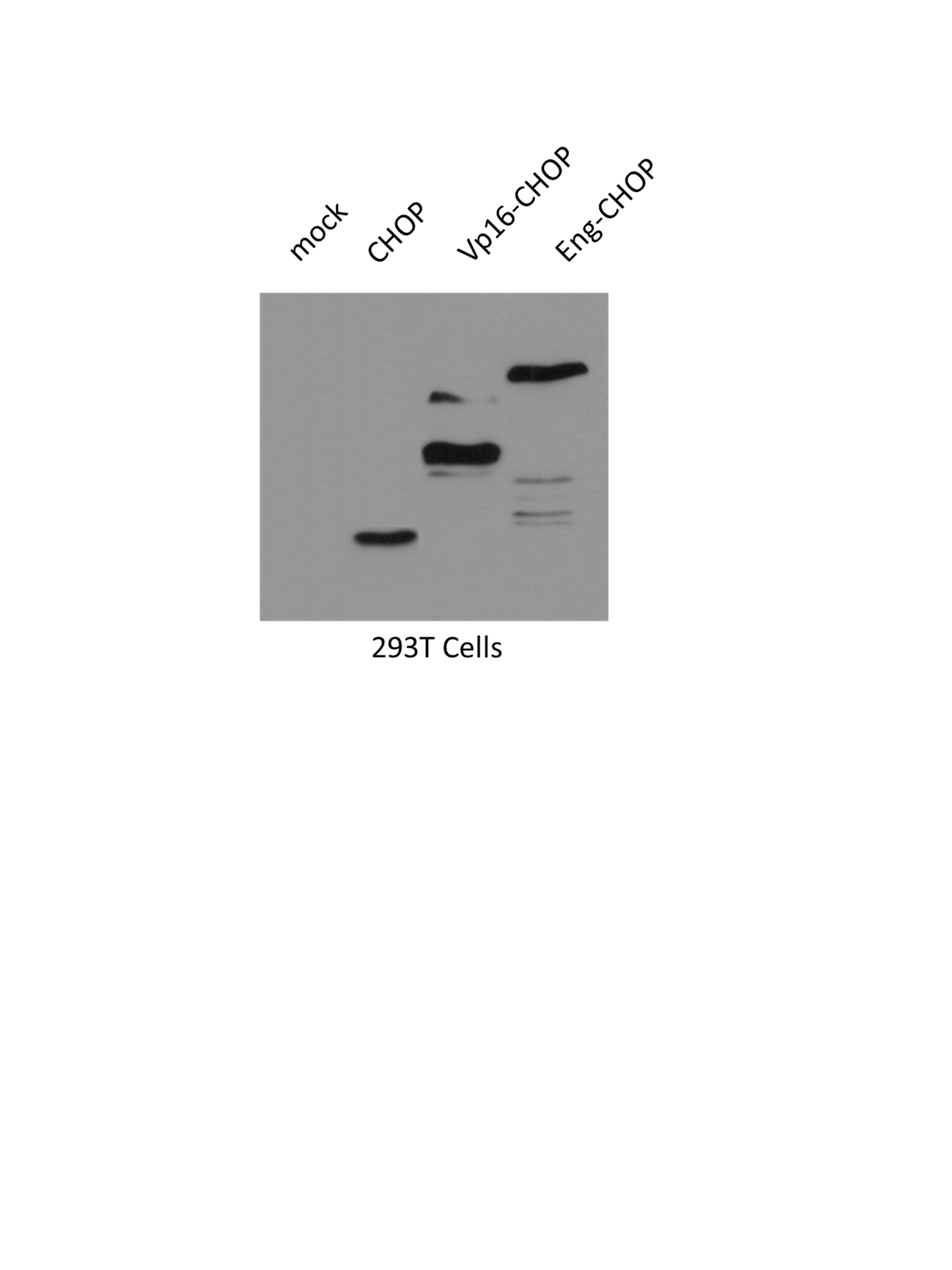

Supplement: Figure S4 — Expression of CHOP chimera proteins. 293T cells were transfected with retroviral expression vectors encoding for wt CHOP, VP16-CHOP and Eng-CHOP. Twenty four hours after transfection, cells were lysed and protein extracts were analyzed by Western blot with anti-CHOP antibodies. (TIF) [file pone.0029498.s004.tif]

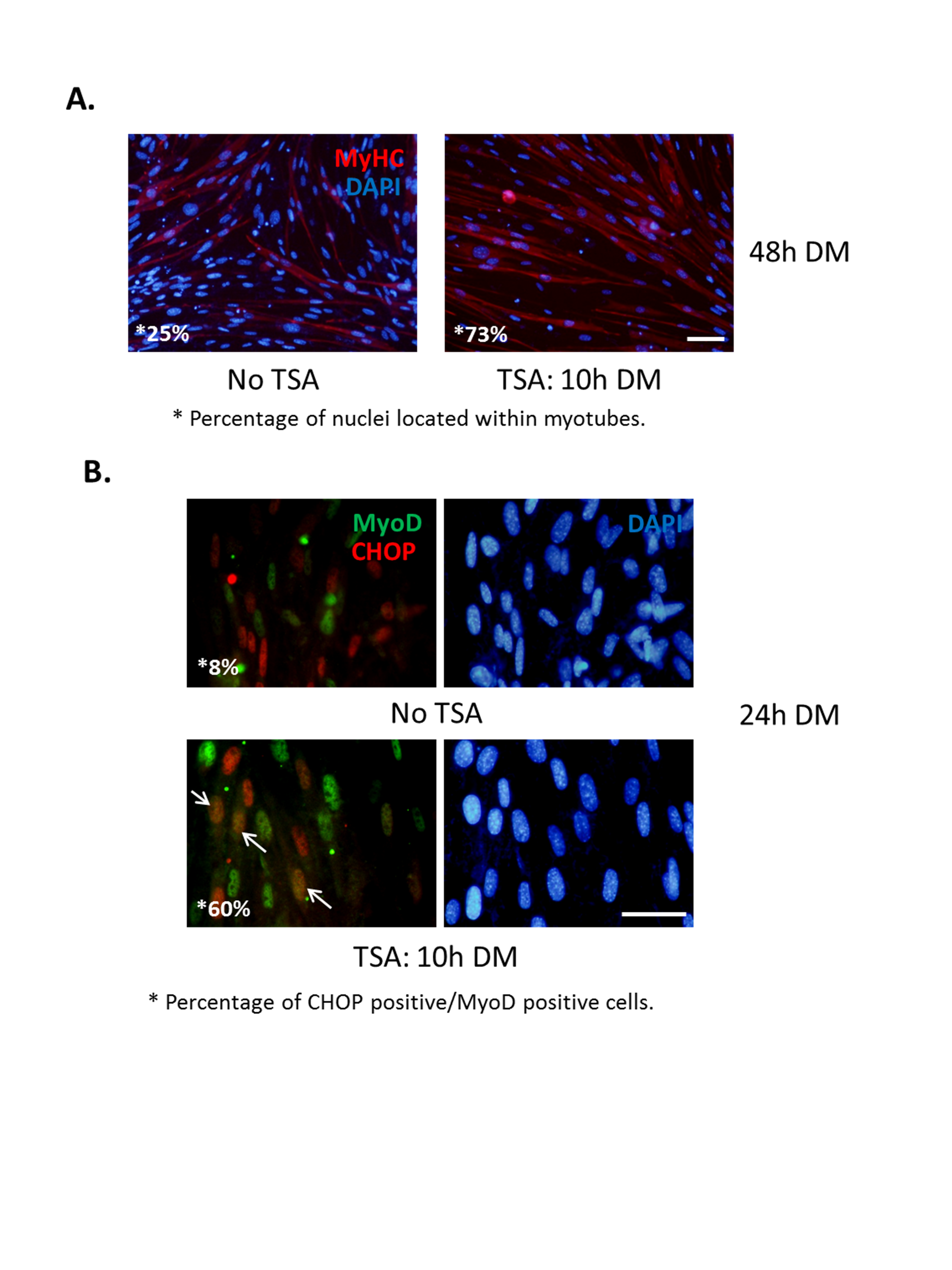

Supplement: Figure S5 — Treatment of C2C12 cells with trichostatin A (TSA) increases co-expression of CHOP and MyoD. C2C12 cells were grown for 10 hours in DM and in the absence or presence of trichostatin A (50 nM). Following that period, medium was replaced by DM. (A) Cells were grown for additional 38 hours (total 48h in DM) and were then immunostained with antibodies against NyHC. Percentage of nuclei within myotubes was calculated from two microscopic fields. Bar, 50 µm. (B) Cells were grown for additional 14 hours (total 24h in DM) and were immunostained with antibodies against CHOP and MyoD. Percentage of CHOP+/MyoD+ nuclei was calculated from 100 CHOP positive nuclei. Bar, 50 µm. (TIF) [file pone.0029498.s005.tif]
